# Get in Touch With Dendritic Epithelial T Cells!

**DOI:** 10.3389/fimmu.2020.01656

**Published:** 2020-07-29

**Authors:** Flavian Thelen, Deborah A. Witherden

**Affiliations:** Department of Immunology and Microbiology, The Scripps Research Institute, La Jolla, CA, United States

**Keywords:** epithelial, DETC, γδT cell, activation, costimulation, epidermis

## Abstract

Innate and adaptive immune systems continuously interchange information and orchestrate their immune responses to protect the host. γδT cells play crucial roles, as they incorporate both innate and adaptive immune characteristics. Dendritic epidermal T cells (DETC) are specialized γδT cells, which are uniquely positioned to rapidly respond to skin wounds and infections. Their elongated dendrite morphology allows them to be in continuous contact with multiple neighboring keratinocytes and Langerhans cells. Cellular interactions are fundamental to the formation, activation and maintenance of immune cell functions during steady state and pathology. Recent technological advances, especially in the field of cellular imaging, have contributed greatly to the characterization of complex cellular interactions in a spatiotemporally resolved manner. In this review, we will highlight the often-underappreciated function of DETC and other γδT cells during steady state and an ongoing immune response. More specifically, we discuss how DETC-precursors are shaped in the fetal thymus during embryogenesis as well as how direct cell-cell interactions of DETC with neighboring epidermal cells shape skin homeostasis and effector functions. Furthermore, we will discuss seminal work and recent discoveries made in the γδT cell field, which have highlighted the importance of γδT cells in the skin, both in humans and mice.

## Introduction

T cell immune responses are tightly connected to their ability to circulate and migrate into tissues, as their priming and effector function is dependent on direct cell-cell interactions. In the last two decades, advancements in imaging techniques, such as two-photon microscopy, have shed light on the complex processes involved in αβT cell priming, effector differentiation and function (1–10). Interestingly, only a handful of studies have used these imaging techniques to study the immune function of γδT cells (11–17). These studies have shown remarkable differences in morphology and migratory behavior of γδT cells residing in different tissues. For example, γδT cells in lymph nodes migrate vigorously (11, 16) in contrast to the slowly migrating γδT cells in the gut parenchyma (14), whereas γδT cells in the epidermis are firmly sessile (15, 18). Moreover, while dermal γδT cells continuously migrate and home to draining lymph node, γδT cells in the epidermis do not recirculate, at least not during steady state conditions (12, 13). Here we describe how γδT cells in the epidermis are formed and, despite their immotile nature, perform their essential guardian function in the biggest barrier tissue. In this review we will focus on the murine epidermal γδT cells, named dendritic epidermal T cells (DETC), which perform essential homeostatic functions and are pivotal for sounding the alarm during an epidermal barrier breach.

## DETC Selection and Seeding of Epidermis

Although in low numbers in secondary lymphoid organs and circulation, γδT cells in both humans and in rodents are concentrated in peripheral organs, such as the digestive tract, lungs or skin (19–21). Further, γδT cells differ from conventional αβT cells, due to their restricted T cell receptor (TCR) diversity (22). Interestingly, when looking at the potential combinations of the variable (V), diversity (D) and joining (J) segments, the γδTCR diversity is significantly higher than both the B cell receptor (BCR) and αβTCR with 10^20^ potential combinatorial diversities, in comparison to 10^11^ and 10^15^, for BCR and αβTCR, respectively (23, 24). Remarkably, the TCR repertoire effectively expressed by γδT cells is greatly limited, with some oligoclonal γδT cell subsets dominating in certain organs. Indeed, γδT cells in mouse epidermis express a very distinct TCR, with most if not all expressing Vγ3-Jγ1-Cγ1/Vδ1-Dδ2-Jδ2-Cδ, Garman nomenclature (25–27). It is worth noting that human tissue-resident γδT cells, at least in skin, gut, and liver, also express a restricted TCR, which is characterized by expression of Vδ1 and distinct from their largely Vδ2-expresssing circulatory counterparts (28, 29). Analysis of the γδTCR structure has revealed a close resemblance to the BCR structure, suggesting the possibility that the γδTCR recognizes antigen directly without the need for MHC-processing and presentation (30). In fact, most γδT cells form normally in beta2-microglobulin knockout mice, which lack MHC-I expression. Further, direct γδTCR binding to pathogen-derived antigens, as well as phospho-antigen, have been reported (31–34). Although in this review we will discuss γδTCR ligands and signaling in the context of epidermal γδT cell development and function, we will not further discuss antigen recognition by the γδTCR, as this has been reviewed recently (35).

DETC are specialized murine epidermis-resident T cells that express the monoclonal Vγ3Vδ1 TCR. DETC precursors seed the epidermis during days 14 through 18 of gestation (20, 26). It was initially thought that since DETC are highly restricted in their TCR usage, Vγ3Vδ1 expression was essential for skin homing. This hypothesis was later rejected as multiple studies have reported DETC formation in different transgenic mouse models, which utilize an array of different Vγ and Vδ gene combinations. Indeed, both Vγ3 and Vδ1 knockout mice develop functional DETC in the epidermis (36, 37). Interestingly, in TCRδ knockout mice, which lack all γδT cells, the epidermis is colonized by αβTCR-expressing DETC (αβDETC), at similar numbers and morphology to conventional DETC. However, αβDETC show reduced functionality and their frequency declines in adulthood (38). Importantly, seeding and colonization of the epidermal niche is restricted to the time from late embryogenesis until a few days after birth, as conditional depletion of DETC in adult mice leads to permanent absence of epidermal T cells, both of γδ and αβTCR origins (39).

Early on, it became clear that γδTCR signaling was essential for DETC development and maintenance, as deletion of molecules involved in TCR signaling, such as ZAP-70, LAT, Syk, or Lck resulted in a significant reduction of DETC (40–43). Indeed, strong TCR signaling in combination with a weak Notch signal is essential for γδT cell linage commitment, whereas low TCR signals together with a robust and sustained Notch signal favor αβT cell linage commitment (44–48). It is noteworthy however, that absence of a Notch signal is not enough to drive γδT cell lineage commitment and that other cellular interactions in the thymus guide γδT cell differentiation (49). A curious observation in a specific FVB mouse strain, shed light on additional factors which drive early stages of DETC formation in the fetal thymus. FVB mice purchased from Taconic (FVB-TAC), but not from Jackson (FVB-JAX), lacked the canonical Vγ3Vδ1 DETC in the epidermis of adult mice. Remarkably, Vγ3Vδ1 DETC precursors were still present in the fetal thymus of these mice yet were found to lack key markers for maturation and skin homing, suggesting a defect in the intrathymic differentiation of these cells (50). A later study pinpointed the defect in DETC formation in FVB-TAC mice to a mutation in the Skint1 gene (selection and upkeep of intraepithelial cells 1) (51). Skint1 is highly expressed in the mouse fetal thymus and in keratinocytes (KC) and overexpression of Skint1 in FVB-TAC mice rescues Vγ3Vδ1 DETC progenitor differentiation (51, 52). However, direct binding of Skint1 to the Vγ3Vδ1 TCR has not been demonstrated. Nonetheless it has been shown that interactions between Skint1^+^ thymic cells and Vγ3Vδ1 cells are essential for imprinting a DETC progenitor phenotype. Indeed, these interactions favor a commitment toward an IFNγ-producing γδT cell fate through the Egr-3 pathway, which inhibits expression of transcription factors favoring IL-17-producing γδT cells, commonly found in the gut lamina propria (53, 54). Interestingly, it became clear that direct TCR signaling is essential to induce such DETC progenitor phenotype as well as to promote the expression of skin homing markers. In fact, TCR signaling induces upregulation of S1PR, CCR10, E-P selectins, and CCR4 (55, 56), as well as upregulation of the transcription factor T-bet (53). The upregulation of these surface markers allows DETC progenitors to egress the thymus via S1PR signaling and to home to the epidermis using the adhesion molecules P- and E-selectin and following the CCR10 ligand CCL27 expressed by KC (55–58). In summary, current evidence suggests that DETC selection, in contrast to the well-described negative selection of αβT cells, is poised for strong self-TCR signaling which promotes expression of skin homing molecules. In humans, the Skint1-like gene is inactive due to multiple premature stop codons (51), therefore, it remains still unknown what requirements are necessary for intrathymic selection and imprinting of human epidermal γδT cell populations. Furthermore, members of the butyrophilin family, the gene family in humans most homologous to the Skints, have been shown to play essential roles in activating circulatory and gut resident γδT cells (33, 34, 59, 60). Therefore, it is possible that a butyrophilin family member could play similar functions as Skint1 for skin γδT cells in humans. However, such equivalent has not yet been described and will need further investigation.

Interestingly, formation and seeding of mouse gut resident CCR9^+^ Vγ5^+^ γδT cells seems to be remarkably biased toward thymocytes that are phenotypically antigen-naïve (61). The distinct TCR-signaling selection of Vγ5^+^ and Vγ3^+^ γδT cells suggest that their TCR-dependent activation in their respective peripheral organs may also be functionally different. Indeed, Vγ5^+^ gut γδT cells are poised to produce IL-17 upon activation, whereas DETC are potent IFNγ producers. However, precisely how this difference in intrathymic selection affects their effector function remains to be investigated.

## TCR Signaling in the Epidermis

**Figure 1:**
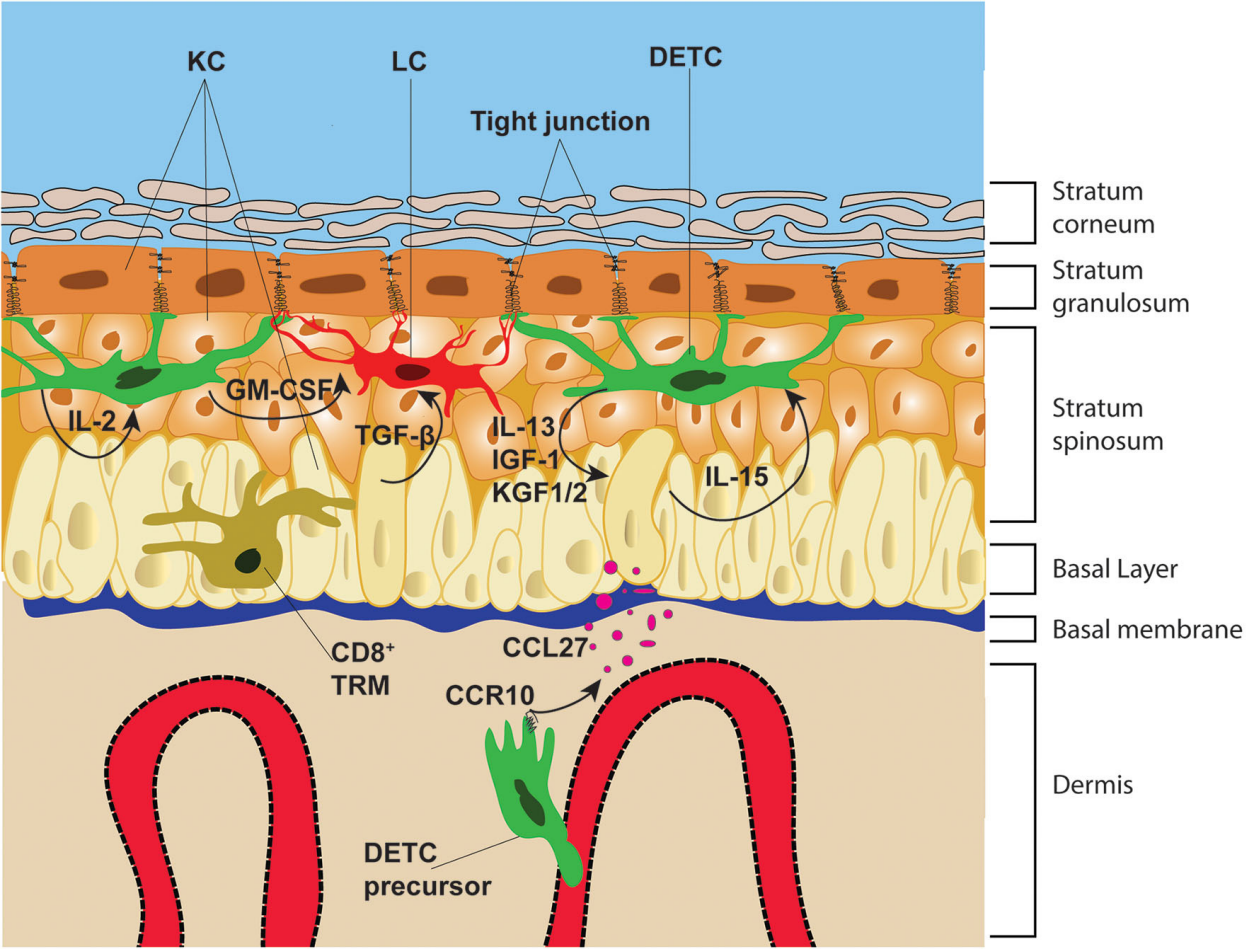
Epithelial architecture and immune cell distribution. The epidermis is divided from the dermis by the basal membrane. The epidermis is composed of four distinct layers: the stratum corneum, stratum granulosum, stratum spinosum and basal layer. These layers are composed mainly of KC that are infiltrated with immune cells, such as DETC, LC, and CD8^+^ TRM. DETC precursors in neonatal mice, home to the epidermis via the blood vessels and following the chemotactic gradient of KC-derived CCL27. DETC and LC form stable interactions with KC tight junctions, whereas CD8^+^ TRM migrate along the basal layer and in-between KC. DETC produce multiple soluble factors that promote KC survival and proliferation and receive IL-15 from KC, which promotes their lifelong survival and self-renewal.

**Figure 2:**
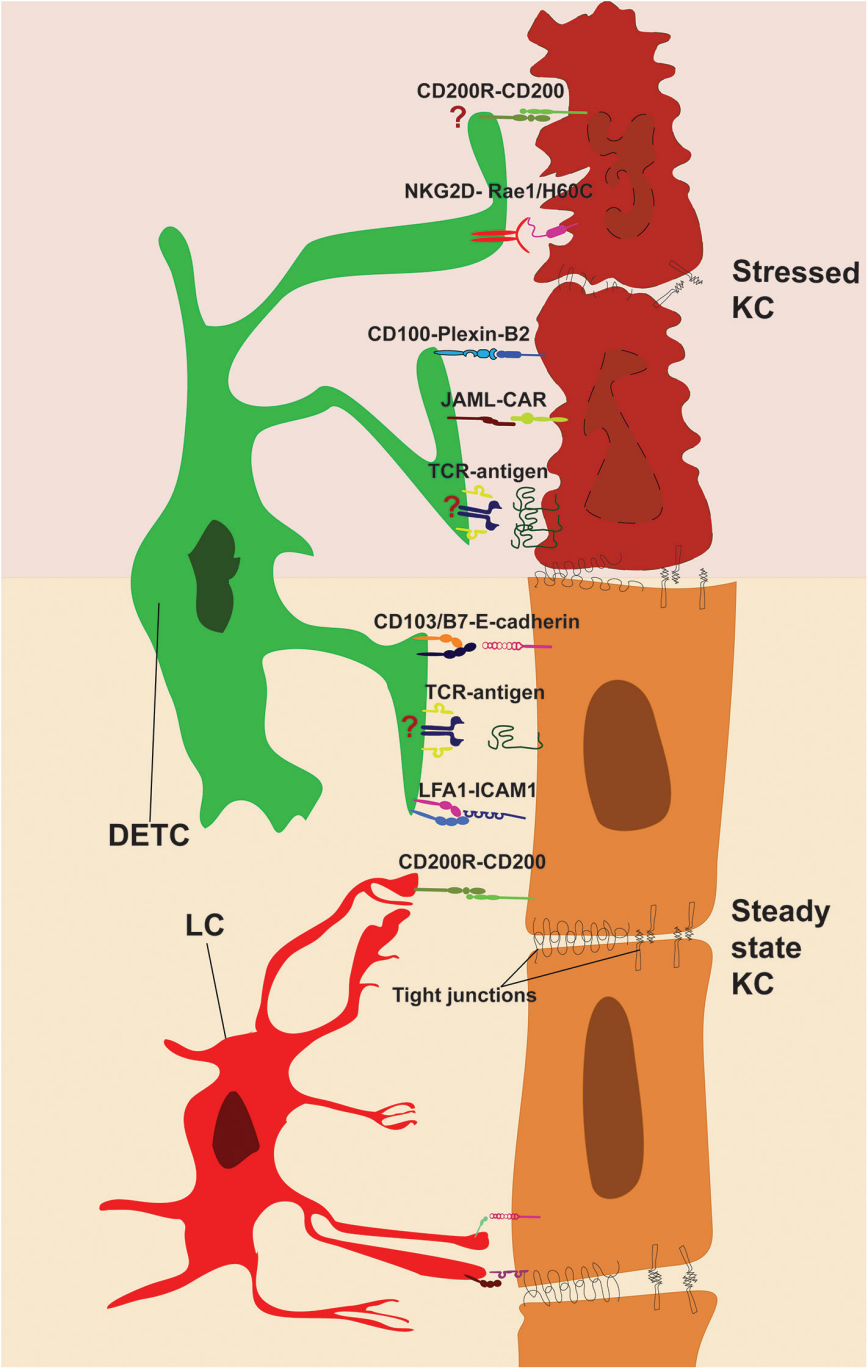
Direct cell-cell contacts regulating immune surveillance of epidermis. DETC and KC form stable interactions during steady state (bottom). TCR and adhesion molecules LFA-1 and CD103 are enriched and engaged with KC-derived ligands at tips of DETC protrusions. LC-KC interaction via CD200R-CD200 suppresses LC activation at steady state. Stressed KC (top) upregulates costimulatory ligands and DETC-specific antigens, which activate DETC via JAML, CD100, NKG2D, and TCR. However, further research is needed to pinpoint the role of antigen presentation and CD200-CD200R signaling in shaping DETC effector functions (indicated by “?”).

The epidermis is the most outer layer of the skin, protecting the organism from environmental elements. The epidermal tissue architecture is orchestrated by differentiating KC, with the top layer containing enucleated KC (stratum corneum), followed by three layers of KC which express tight junction molecules, such as ZO-1 and Claudin-1 (62–64), referred to as stratum granulosum. Under the stratum granulosum are located the stratum spinosum and the basal layer, which are bound to the thick and complex basement membrane that separates the epidermis from the dermis. Immune cells, such as γδT cells, Langerhans cells (LC) and tissue resident memory (TRM) CD8^+^ T cells are situated in the basal layer and stratum spinosum. DETC are the exclusive T cell subset in the epidermis of naïve rodents (65), whereas in humans, both αβT cells and γδT cells make up the T cell compartment of the epidermis, and both T cell subsets seem to have somewhat parallel functions to mouse DETC (66). As the name indicates, DETC are characterized by their arborized and elongated cell body (67), which remarkably resembles LC morphology. In contrast, CD8^+^ TRM, which have elongated cell bodies in comparison to T cells in lymph nodes, form significantly less cellular protrusion than DETC in the epidermis (15). Notably, whereas CD8^+^ TRM retain migratory capability in the epidermis at steady state, both DETC and LC are mainly sessile and only slowly and seldomly retract and extent their protrusions (15, 18). The elongated dendrites of both DETC and LC are orientated upwards toward the stratum granulosum (18, 64, 68) where they interact with KC tight junctions. Moreover, DETC protrusions are simultaneously in stable contact with multiple KC and LC (Figure 1). This is in stark contrast with CD8^+^ TRM dendrites which are continuously contracting and extending, protruding laterally between KC in the basal layer probing the surrounding cells for cognate antigen (69). The highly stable DETC-KC and LC-KC contacts at steady state elude to the continuous monitoring function of these cells, ensuring barrier integrity. Unexpectedly, it has been shown that the γδTCR, together with the integrins CD103 and LFA1, is highly enriched at the tips of the dendrites and actively engages at tight junctions of KC (Figure 2). Indeed, constitutive γδTCR signaling during steady state has been reported in DETC by staining for phospho-tyrosine 142 of the CD3ζ chain (18). This constitutive TCR-activation seen in Vγ3Vδ1 DETC, but not in naturally occurring Vγ3^neg^- or αβDETC, suggests that self-antigen specific interaction is occurring during steady state. The study from Minagawa et al. (70), further strengthens this idea, showing that during early embryonic development Vγ3^+^ cells can also be found in the gut epithelia, yet only the skin epidermal Vγ3^+^ cells persist in newborn mice suggesting Vγ3Vδ1 self-antigen recognition in epidermis promotes DETC persistence. Nevertheless, it is important to highlight that a DETC-specific antigen could not be detected by Vγ3Vδ1-tetramer staining in resting skin, whereas KC-derived antigen was readily detected near skin wound edges (71), suggesting antigen upregulation following KC damage. However, lack of DETC-tetramer staining at steady state, could be explained by low antigen abundance and/or accessibility in non-wounded skin.

Several studies have shown the paramount importance of TCR signaling for DETC formation, maintenance, self-renewal and activation upon barrier disruption (38, 40–43, 50). For instance, deficiency in LAT, a linker protein for TCR signaling, inhibits DETC proliferation during neonatal epidermal colonization (72). Further, LAT conditional depletion in adult mice, reduced DETC homeostatic proliferation, underlining the importance of constant TCR engagement for DETC self-renewal (72). Studies using transgenic KN6 mice, expressing the Vγ2Vδ7, further expand on the hypothesis of a requirement for balanced TCR signaling supporting DETC maintenance and survival. The KN6 γδTCR has been shown to recognize two non-classical MHC-I molecules T10 and T22, which are highly expressed in the skin (73). These mice possess similar numbers of DETC at a young age (74), however their numbers decrease over time (58). The authors argue that the defect in KN6 DETC maintenance is caused by sustained strong TCR stimulation, which leads to excessive DETC activation in the epidermis. Indeed, KN6 DETC numbers were rescued in KN6 mice deficient for interleukin-2-inducible T-cell kinase (ITK), a TEC family kinase involved in TCR signaling. Interestingly, polyclonal ITK knockout mice showed a decrease in DETC numbers, which was linked to shortcomings of positive selection in fetal thymus and failure to upregulate skin homing markers in DETC progenitors (58). In sum, the DETC TCR appears to be constitutively active at low levels during steady state, promoting a basal level of DETC activation essential for their maintenance. Yet, this appears to be below a threshold required for full activation and effector function of DETC.

## DETC Residency and Homeostatic Function in Epidermis

Lifelong persistence of DETC and their remarkable uniform distribution in the epidermis, indicates their ability to sense and fill empty spaces via proliferation. The self-renewal mechanism is partially regulated by TCR signaling, as discussed above (58, 72, 75), as well as by soluble factors, such as IL-15 produced by surrounding KC (76) (Figure 1). Defects in IL-15 downstream signaling in mice lacking JAK3 or STAT5, have clearly shown the pivotal role of IL-15 in promoting DETC proliferation and survival (77, 78). These signals seem to be particularly important during the early expansion of DETC precursors in newborn mice. Indeed, early studies suggested that DETC precursors quickly proliferate in neonatal epidermis and expand laterally until covering the entire epidermal surface (67). This has been recently further characterized, utilizing multicolor fate mapping, illustrating how founder DETC, which seed the epidermis during embryogenesis, proliferate laterally forming clusters of clones (79). The expression of the stem-cell marker c-Kit has been linked to the early expansion of DETC founders in neonatal skin. Mice lacking the expression of the Aryl hydrocarbon receptor (Ahr) show reduced expression of c-Kit in newborn epidermal DETC. Ahr^−/−^ DETC fail to expand and colonize the epidermis, leading to complete loss of DETC in adult mice. In adult Ahr^−/−^ animals, the epidermis is not occupied by αβDETC likely due to the restricted temporal seeding window as aforementioned (80). Performing longitudinal imaging, the process of self-renewal has been visualized *in vivo*, DETC sense empty spaces in the surrounding tissue, by mechanisms that are not yet fully understood, and quickly proliferate. The daughter cell actively migrates in a directional fashion toward the empty niche and establishes residency (18). Similarly, DETC surrounding a skin graft show slow expansion and directional immigration into grafted tissue (79). It would be interesting to further study the colonization of host DETC into skin grafts that are depleted of donor DETC and see if their immigration is accelerated compared to non-depleted donor skin grafts, indicative of their ability to sense empty niches. Moreover, chemical inhibitors or genetic modification of donor skin could be used to pinpoint factors promoting DETC proliferation and lateral expansion. These findings could reveal new therapeutic approaches for improving treatment of chronic wounds, which are prevalent in diabetic and obese patients.

It has become clear that metabolic alteration, both in humans and mice, can have detrimental effects on skin regeneration, which can lead to chronic non-healing wounds and life-threatening complications (81–84). Interestingly, epidermal γδT cell numbers and functions are decreased in these conditions. For example, hyperglycemia inhibits DETC proliferation as well as their responsiveness to skin injuries, which is linked to the reduced activity of the master regulator mTOR as well as the lower level of active phosphorylated STAT5 (85). Similar defects of DETC functions have been described in mice receiving prolonged treatment with the mTOR inhibitor rapamycin (86). Rapamycin dampens the production of IL-15 in KC, which leads to a decrease in DETC numbers and in their production of the KC growth factor IGF-1. Local administration of either IL-15 or IGF-1 can rescue DETC proliferation and functions, promoting wound healing (86–88). Recent, studies have further shown that nutrition can have a significant impact on γδT cell functions and proliferation. A ketogenic diet, which consists of a low carbohydrate and high fat content diet, boosts γδT cell proliferation in lung, which can be beneficial during influenza infection (89). However, prolonged non-caloric-restricted ketogenic diet causes weight gain and obesity in mice, reversing the beneficial effects on γδT cells and augmenting inflammation (90). Exactly how diet and its metabolites can modulate γδT cell proliferation, and if these factors affect all γδT cell subsets in the body, is still unknown. Further, it will be interesting to investigate if caloric restriction in ketogenic diet could promote γδT cell function in the long term and if this could be beneficial in certain disease models, such as wound healing, inflammatory bowel diseases and cancer.

## Costimulation and Direct Cell-Cell Interactions

Epidermal γδT cell activation during skin injury is important to regulate immune responses and favors rapid wound healing both in humans and mice (66, 91). As mentioned above, DETC-tetramer staining in wounded epidermis detects rapid appearance of a KC-derived DETC-specific antigen (71). Antigen recognition and TCR-induced activation of DETC seem to be important for wound healing processes, since αβDETC in TCRδ^−/−^ animals do not sense or respond to KC-derived antigen, which leads to a delay in wound healing (91). It is noteworthy that, although direct binding to the TCR has not been reported, KC at wound edges express multiple members of the Skint family. Decreased expression of Skint family members in aged mice has been linked to reduced STAT3-phosphorylation compared to young mice. Selective downregulation of STAT3 or Skint genes affects DETC activation and delays wound healing (92), suggesting that Skint family members play not only important roles in regulating DETC selection in fetal thymus but are also involved in DETC activation in epidermis.

It has been suggested that DETC positive selection in the thymus ensures that DETC have a high activation threshold in the epidermis. Indeed, like what has been described for αβT cells, DETC activation is not solely due to TCR signaling but requires synergy with costimulatory receptors. Stressed KC at wound edges upregulate multiple surface molecules that can promote DETC activation. Semaphorin 4D (also known as CD100) is a receptor for Plexin-B2, Plexin-B1, and CD72 and it is expressed by multiple immune cells, including DETC. PlexinB2-CD100 interactions were originally described as providing axon-guidance cues, yet ligation of CD100 and its role outside the nervous system are now evident (93, 94). During skin injury, Plexin-B2 is rapidly upregulated and expressed on the surface of KC at wound edges and its interaction with CD100 on DETC induces bidirectional signaling. Activation via CD100 induces DETC rounding and secretion of IL-2. Indeed, CD100-deficient mice show delays in wound healing (95). Furthermore, subcutaneous injection of soluble CD100 promotes wound healing in a diabetic mouse model (96). On the other end, CD100 interaction with Plexin-B2 promotes the NF-κB signaling pathway in KC, which leads to secretion of pro-inflammatory molecules, such as IL-1β. Knockdown experiments have shown that inhibiting Plexin-B2 expression in KC can dampen inflammation and reduce pathogenesis in a murine psoriasis model (97). Similarly, CD100-induced inflammation has been linked to the promotion of contact hypersensitivity (CHS), caused by excess immune cell infiltration (94). Thus, CD100 is a strong costimulatory receptor that facilitates DETC activation, leads to DETC rounding and promotes inflammatory immune responses.

Similar upregulation of stress molecules in KC have been reported for the NKG2D ligands (Figure 2), such as retinoic acid early-inducible 1 (Rae-1) isoforms, murine UL-16 binding protein-like transcript 1 (Mult1) and histocompatibility 60 (H60). These ligands are absent or expressed at low level at steady state and become upregulated in stressed, chemically irritated cells and following DNA damage (98–100). Indeed, skin exposure to carcinogens induces H60C expression in KC and leads to NKG2D-dependent DETC activation (101). Similarly, 2B4, a non-MHC-dependent surveillance receptor on DETC, has been reported to prevent cancer formation by lysis of transformed cells (102). Additionally, Strid et al. (103) found that overexpression of Rae1 specifically on KC, was sufficient to induce NKG2D-dependent DETC activation and secretion of IL-2 and IL-13. Interestingly, LC which do not express NKG2D, were activated during the same timeframe, suggesting a rapid DETC-dependent tissue-wide state of alert. Indeed, activated DETC produce a variety of inflammatory molecules, such as CCL5 (also known as RANTES), which is a potent activator of dendritic cells (104, 105). NKG2D-H60C interaction has been shown to promote wound healing, as blocking antibodies against H60C reduced DETC rounding and delay wound healing (106).

Finally, junctional adhesion molecule JAML which binds to the Coxsackie and adenovirus receptor (CAR) has been identified as a potent costimulatory receptor of DETC and γδ intraepithelial lymphocytes (IEL) in the gut. JAML expression is upregulated in DETC upon activation and its ligation to CAR induces recruitment of PI3K to the intracellular domain of JAML, as delineated for the αβT cell costimulatory receptor CD28 (107). Potent costimulation via JAML could be detected in DETC and γδ IEL but not in circulating γδT cells, further underlining the tissue-specific adaptation of γδT cells. Furthermore, blocking CAR-JAML interactions delays wound healing at a rate comparable to that of TCRδ knockout mice (108). To equilibrate the effects of costimulatory molecules, KC express constitutively CD200. Its receptor CD200R is a potent inhibitor of LC activation, which prevents autoimmune reactions during steady state (109). Interestingly DETC upregulate CD200R expression upon activation, however it is yet to be elucidated what inhibitory function, if any, CD200R may play in the context of wound healing.

As previously mentioned, DETC activation is characterized by rounding and loss of arborized morphology in these cells, which has been suggested to allow DETC migration as evidence suggests they accumulate at the wound edge (91). Supporting this hypothesis, activated DETC downregulate adhesion molecules, such as CD103 and E-cadherin, which have been linked to cell residency both in DETC and TRM, as well as promoting LC egress (38, 69, 110–113). In contrast, Occludin, a transmembrane enzyme normally found at tight junctions, is upregulated in activated DETC. Its expression has been associated with DETC rounding and with the ability of DETC to egress and home to draining lymph nodes (114). Similarly, DETC migration to, and accumulation in, skin draining lymph nodes has been reported in a CHS study (115). However, these studies did not directly visualize DETC migration, therefore it is yet to be directly demonstrated whether, upon activation, DETC gain cell motility and actively migrate toward the draining lymph node or wound edge.

## DETC Function Upon Activation

As aforementioned, DETC TCRs are continuously engaged. This basal activation allows DETC to secrete low-levels of the IL-2 family member IL-13 at steady state. IL-13 promotes homeostatic KC proliferation and survival, which is disrupted in IL-13-deficient mice, causing increased susceptibility to carcinomas (116). In fact, it has become clear that both in humans and mice, γδT cells play an important part in regulating homeostatic functions of organs as well as in promoting tissue repair upon injury (66, 91, 117, 118). DETC produce a multitude of cytokines and growth factors, that modulate neighboring cell functions as well as stimulating themselves via autocrine signaling. For instance, KGF-1 and KGF-2 (also known as FGF-7 and -10), produced by DETC, promote KC proliferation, maturation and migration. Furthermore, DETC-derived KGF-1 and -2 induce epithelial cell production and secretion of hyaluronan, which significantly augments neutrophil and macrophage infiltration at skin wounds (119). The secretion of these growth hormones by activated DETC is essential for rapid wound healing (91, 120). Insulin-like growth factor (IGF-1) is another important soluble factor produced by DETC during steady state (121) as IGF-1 regulates KC development and maintenance (122). Further, IL-15 secreted by KC promotes IGF-1 production by DETCs, generating a positive feedback loop (88) (Figure 1). Interestingly, although in humans there is not a direct counterpart of DETC, human epidermal γδ and αβ T cells also produce IGF-1, supporting wound healing. In chronic wounds this beneficial production of IGF-1 is absent. Indeed, isolated γδ and αβ epidermal T cells from such wounds are completely unresponsive to stimulation, indicative of their essential function in regulating wound-healing and resembling the functions of mouse DETC (66). Finally, DETC are the main source of GM-CSF in the epidermis, which has been shown to promote LC maturation (123). Indeed, in Ahr-deficient mice which lack DETC, GM-CSF levels in the skin were reduced causing defects in LC maturation and activation (80, 124). Interestingly, although DETC are developmentally poised to produce IFNγ (53), a subset of DETC produce IL-17a, which is essential for rapid wound healing (125). Whether this IL-17 production ability is also imprinted in the thymus or is a product of the spatiotemporal activation of this population of DETC is yet to be determined.

## Conclusions

DETC are strategically positioned to survey and rapidly respond to a pathogenic insult or a mechanical disruption of the barrier tissue. Their characteristic morphology, as well as their static migratory behavior, are indicative of their continuous surveillance function. In fact, their importance in sensing and responding to skin injuries has been reported both in humans and mice (66, 91). Although in recent years significant advancements have been made in understanding DETC biology, many aspects on how their direct cell-cell contact with neighboring cells regulates their homeostatic function and allows for rapid activation upon injury, remain to be elucidated.

We would like to dedicate this review to Professor Wendy Havran, who was a pioneer of the DETC research field, and we had the pleasure to work with. The sad and sudden loss of this wonderful immunologist and mentor has shocked the γδT cell field. Her seminal work, such as describing the unique developmental waves of DETC (20) and their importance in mediating wound healing (91), has sparked worldwide interest and research in these unconventional T cells. We know that her legacy has and will further fuel new discoveries in this exciting research field.

## Author Contributions

FT and DW wrote the manuscript. All authors contributed to the article and approved the submitted version.

## Conflict of Interest

The authors declare that the research was conducted in the absence of any commercial or financial relationships that could be construed as a potential conflict of interest.
